# Early-Released Interleukin-10 Significantly Inhibits Lipopolysaccharide-Elicited Neuroinflammation In Vitro

**DOI:** 10.3390/cells10092173

**Published:** 2021-08-24

**Authors:** Yubao Wang, Pei Yu, Yi Li, Zhan Zhao, Xiaomei Wu, Lu Zhang, Jing Feng, Jau-Shyong Hong

**Affiliations:** 1 Institute of Infectious Diseases, The Second Hospital of Tianjin Medical University, Tianjin 300211, China; 18822024074@163.com (P.Y.); wuxiaomei718@163.com (X.W.); 2 Neurobiology Laboratory, National Institute of Environmental Health Sciences, National Institutes of Health, Research Triangle Park, NC 27709, USA; lisalie5252@hotmail.com (Y.L.); zhaozhan726@163.com (Z.Z.); zhangluzzuedu@163.com (L.Z.); hong3@niehs.nih.gov (J.-S.H.); 3 Respiratory Department, Tianjin Medical University General Hospital, Tianjin 300052, China

**Keywords:** microglia, interleukin-10, lipopolysaccharide, neuroinflammation, tumor necrosis factor alpha, interleukin-1β, β2-adrenergic receptor, Arg-1

## Abstract

Anti-inflammatory cytokine interleukin (IL)-10 is pivotal for limiting excessive inflammation in the central nervous system. Reports show that lipopolysaccharide (LPS)-induced microglial IL-10 emerges in a delayed manner in vitro and in vivo, lagging behind proinflammatory cytokines to facilitate the resolution of neuroinflammation. We hypothesized that IL-10 releases quite quickly based on our pilot investigation. Here, we uncovered a bimodal expression of microglial IL-10 gene transcription induced by LPS in mouse primary mixed glial cultures. This pattern consisted of a short brief early-phase and a long-lived late-phase, enabling the production of IL-10 protein in a rapid manner. The removal and addition of IL-10 protein assays indicated that early-released IL-10 exerted potent modulatory effects on neuroinflammation at picomolar levels, and IL-10 released at the onset of neuroinflammation is tightly controlled. We further showed that the early-released, but not the late-released, IL-10 was crucial for mediating and potentiating the anti-inflammatory function of a β2-adrenergic receptor agonist salmeterol. This study in vitro highlights the essential role of early-released IL-10 in regulating the appropriate degree of neuroinflammation, overturning the previous notion that microglial IL-10 produces and functions in a delayed manner and providing new insights into anti-inflammatory mechanisms-mediated neuroimmune homeostasis.

## 1. Introduction

Neuroinflammation plays a critical role in a variety of infectious and non-infectious disorders in the central nervous system (CNS), ranging from neurodegenerative diseases to mental disorders [1,2]. Dysregulated or persistent activation of microglia, the main immune cell in the brain, may lead to chronic low-grade neuroinflammation and neuronal damage [3]. One of the most important mechanisms limiting the detrimental effects of excessive neuroinflammation is to enhance the production of a potent anti-inflammatory cytokine interleukin-10 (IL-10). IL-10 is known to suppress the production of proinflammatory mediators through several mechanisms, such as destabilizing proinflammatory cytokine mRNA, negatively regulating Toll-like receptors (TLRs) signaling transduction, and inhibiting inflammasome activation [4,5,6]. In addition, IL-10 can protect tissues during inflammation by facilitating wound repair signaling [7,8].

Molecular mechanisms regulating IL-10 expression by peripheral immune cells were well studied [5,9,10]. By contrast, much less is known regarding the modulation of IL-10 expression in the CNS. Although astroglia can produce IL-10 in response to ligands acting on TLR3 receptor [11], several investigations in vitro and in vivo indicated that microglia are the main source of IL-10 production in the CNS upon the stimulation of TLR4 receptors [12,13,14,15]. Double immunofluorescence staining showed that IL-10 expression was localized in microglia in vivo after lipopolysaccharide (LPS) injection [13]. Our previous report, using three different primary cultures (neuron-enriched, astroglia-enriched, and microglia-enriched cultures), showed that LPS-induced mRNA transcription and extracellular secretion of IL-10 and TNFα occurred only in microglia and not astroglia or neurons [12]. Our findings were supported by reports from other labs [13,15]. Similarly, it was documented that treatment of mixed glial cell cultures with either LPS or a cytokine mix increases the expression of IL-1β in microglia but not in astroglia [16].

Time course studies indicate that IL-10 production rises at later stages of neuroinflammation, lagging behind the release of most proinflammatory factors [12,17]. Several studies in vitro or in vivo pointed out that isolated microglia produce IL-10 in a delayed manner with elevated IL-10 mRNA expression observed at about 8 h and detectible protein release at about 24 h later after stimulation of microbial pathogens or TLR ligands [13,18,19,20,21]. The delayed release of IL-10 suggests that the major roles of this cytokine are immune resolution and tissue repair [22,23].

Our lab has recently reported that intranigral injection of LPS produced much higher levels of mRNA of IL-1β, Nod-like receptor protein 3 (NLRP3), and caspase-1 in the midbrain of IL-10^−/−^ mice than WT mice measured at just 6 h after the injection [24]. These findings indicated that the deficiency of IL-10 aggravates LPS-elicited neuroinflammation and suggests dual roles of IL-10 in the modulation of brain inflammation. In addition to facilitating the resolution of neuroinflammation, we hypothesized that early release of IL-10 might play a critical role in subduing LPS-initiated acute inflammation. The main purpose of this study was, therefore, to determine whether LPS elicits the early release of IL-10 using primary midbrain cell cultures and investigate the functional role of this cytokine in regulating LPS-induced acute neuroinflammation.

Here, we described that LPS enhanced the IL-10 mRNA level in a bimodal fashion, consisting of a short early-phase followed by a long-lasting late-phase in primary mixed glial cultures. An increase in supernatant IL-10 protein was detected as early as 3–4 h after LPS stimulation. Although the amount of early-released IL-10 was at picomolar levels, it exerts potent inhibition on the production of TNFα and IL-1β. To further elucidate the potential roles of early-released IL-10 in the acute phase of inflammation, salmeterol, a long-acting β2-adrenergic receptor agonist that can cross the blood–brain barrier such as clenbuterol, was used [25,26]. Salmeterol, a bronchodilator, was found directly inhibiting microglial proinflammatory cytokines and amplifying microglial IL-10 expression [26,27]. We found that salmeterol potentiated early-phase but not late-phase induction of IL-10 mRNA and protein, suggesting that early-released IL-10 might play a crucial role in mediating salmeterol-elicited anti-inflammatory effect. Moreover, genetic or pharmacological inhibition of IL-10 hampered both proinflammatory cytokine repression and Arg1 enhancement by salmeterol. In summary, this study highlights the novel bimodal transcription of the IL-10 gene and demonstrates LPS-induced early-released IL-10 plays a key role in modulating neuroinflammation in vitro.

## 2. Materials and Methods

### 2.1. Animals

Male C57BL/6J and IL-10^−/−^ mice at the age of 10 to 12-week-old were purchased from The Jackson Laboratory (Bar Harbor, ME, USA). All breeding and housing procedures and experimental protocols were authorized by IACUC (Institutional Animal Care and Use Committee, Bethesda, MD, USA) of NIH (National Institutes of Health, Bethesda, MD, USA).

### 2.2. Reagents

LPS (E. coil O111:B4) used for cell culture studies was purchased from Calbiochem (San Diego, CA, USA; cat# 437627), and for animal studies, it was purchased from Sigma-Aldrich (St. Louis, MO, USA; cat# L3012). IL-10, tumor necrosis factor alpha (TNFα), and interleukin-1β (IL-1β) enzyme-linked immunosorbent assay (ELISA) kits and salmeterol (a long-acting selective β2-adrenergic receptor agonist) were obtained from R&D Systems (Minneapolis, MN, USA). Recombinant mouse IL-10, Ultra-LEAF™ Purified anti-mouse IL-10 antibody, and isotype IgG were from Biolegend (San Diego, CA, USA). The anti-mouse IL-10 antibody from Biolegend was used as the detecting/capture antibody for ELISA/ enzyme-linked immunospot (ELISPOT) assay and for neutralization of mouse IL-10 bioactivity in vivo and in vitro (https://www.biolegend.com/en-us/products/ultra-leaf-purified-anti-mouse-il-10-antibody-17764, accessed on 6 August 2021). The specificity and utility of this antibody were further validated in our laboratory. We found that enhanced IL-10 protein level was detected in the supernatant of LPS-stimulated mouse primary cell cultures by the R&D IL-10 ELISA kit, but no significant difference between vehicle and treatment with LPS plus anti-mouse IL-10 antibody was observed.

### 2.3. Primary Mouse Mixed Glial Cultures

Primary mixed glial cultures containing about 30% microglia and 70% astroglia were prepared from mouse pups as previously described [28]. Whole brains of postnatal day 1 neonates of C57BL/6J or IL-10^−/−^ mice were dissociated by trituration in DMEM/F12 media after stripping blood vessels and meninges. Cells were plated to poly-d-lysine-coated 24-well (5.5 × 10^5^/well) plates with 0.5 mL/well of DMEM/F12 mixed glial culture media and maintained in a humidified 37 °C, 5% CO_2_ incubator. The medium was refreshed every 3 days with 1 mL/well of mixed glial culture media (DMEM/F12). Cultures were available for treatment 14 days after initial seeding. The mixed glial cultures contained microglia and astrocytes. The reasons why we used mixed glial cultures instead of enriched microglia were described in detail in Section 3. The effects of LPS on cytokines expression in mixed glial cultures serve as proof-of-concept experiments. However, it should be noted that the significance of this in vitro finding in clinical settings is not presently clear.

### 2.4. Real-Time RT-PCR Analysis

Total RNA was extracted from cell cultures or mouse brain tissues with QIAGEN RNeasy Mini Kit (Valencia, CA, USA) to detect mRNA levels of TNFα, IL-10, IL-1β, and Arg1 according to the previous description [29]. Total RNA was reversely transcribed with MuLV reverse transcriptase and oligo-dT primers followed by real-time PCR analysis. The primer sequences were as before [29]. SYBR Green PCR Master Mix and QuantStudio 6 Flex Real-Time PCR System (Applied Biosystems, Foster City, CA, USA) were utilized for real-time PCR amplification according to the manufacturer’s protocols. Amplifications were performed at 95 °C for 10 s, 55 °C for 30 s, and 72 °C for 30 s for 40 cycles. All samples were measured in duplicate and normalized with GAPDH using the 2^−ΔΔCt^ method. Fold changes in each treatment were normalized to the vehicle group at the 0 timepoint as 1 unit.

### 2.5. Cytokine ELISAs

IL-10, TNFα, and IL-1β protein concentrations were measured in culture supernatants in accordance with the manufacturer’s instructions by using enzyme-linked immunosorbent assay (ELISA) kits from R&D Systems, respectively. Colorimetric assays were quantified with a spectrophotometer.

### 2.6. Statistical Analysis

Data were presented as the mean ±SEM. One-way ANOVA followed by Bonferroni post hoc multiple comparison test was used for comparison of more than two groups, and two-way ANOVA followed by Bonferroni post hoc multiple comparison test was used for comparisons of more than two parameters, respectively. Data were analyzed by Prism (v7.00, GraphPad, San Diego, CA, USA). The *p*-values less than or equal to 0.05 were regarded statistically significant.

## 3. Results

### 3.1. Bimodal Expression of Microglial IL-10 mRNA upon LPS Stimulation

**Figure 1:**
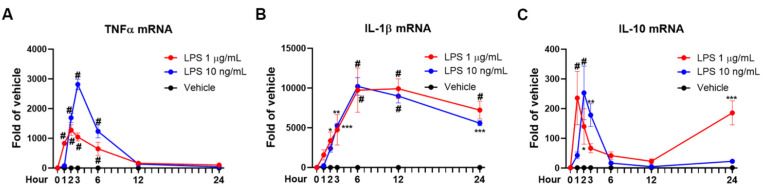
Microglial anti-inflammatory cytokine IL-10 mRNA peaks earlier than proinflammatory cytokines with a bimodal fashion upon LPS stimulation in vitro. Relatively increased mRNA levels of TNFα (**A**), IL-1β (**B**), and IL-10 (**C**) were measured by qPCR at 1 h, 2 h, 3 h, 6 h, 12 h, and 24 h after LPS (10 ng/mL or 1 µg/mL) treatment in cell cultures. Results were from 3 independent experiments performed. * *p* < 0.05, ** *p* < 0.01, *** *p* < 0.001, and # *p* < 0.0001 compared with vehicle group. Two-way ANOVA followed by Bonferroni post hoc multiple comparison test.

We performed a time-course study to measure IL-10 mRNA levels in mixed glial cultures at 1, 2, 3, 6, 12, and 24 h after LPS treatment. Upon LPS at 1 µg/mL stimulation, the increase in IL-10, TNFα, and IL-1β mRNA peaked around 1 h, 2 h, and 6 h, respectively. It was interesting to observe that anti-inflammatory cytokine IL-10 mRNA expression peaked earlier than proinflammatory cytokine TNFα and IL-1β (Figure 1A–C). The mRNA half-life was approximately 1.5 h for IL-10, 6–7 h for TNFα, and more than 18 h for IL-1β. This early rise of IL-10 mRNA declined rapidly, and its half-life was the shortest among detected cytokines. Interestingly, the second phase of IL-10 mRNA expression appeared after 12 h and continued to rise till 24 h after LPS treatment.

Bimodal increase in IL-10 mRNA expression was observed by both doses of LPS, which is different from the pattern of both TNFα and IL-1β (Figure 1). Low dose (10 ng/mL) and high dose (1 ug/mL) of LPS produced similar time course and magnitude of early-phase IL-10 mRNA expression (Figure 1C). IL-10 mRNA of the LPS high dose group peaked a little bit earlier (at 1 h) than that of low dose (at 2 h). The appearance of the early peak of IL-10 expression occurred about the same time or earlier than that of TNFα, but far much earlier than that of IL-1β (Figure 1). The late-phase IL-10 mRNA levels produced by LPS at 10 ng/mL were significantly lower than those by LPS at 1 µg/mL (Figure 1). Thus, LPS caused a bimodal transcription expression of the IL-10 gene with a transient dose-independent early-phase and a long-lasting dose-dependent late-phase. To the best of our knowledge, this unusual early and short-lived expression of IL-10 mRNA upon LPS stimulation has not previously been documented. 

The reasons for utilizing primary mixed glial cultures which contain both microglia and astroglia in this study are two-fold: (1) enriched microglia are known to be semi-activated, and the presence of astroglia can stabilize microglia [30]; (2) LPS fails to increase IL-10 expression in astroglia-enriched cultures and microglia is the major contributor as mentioned in the Section 1. We previously indicated the non-responsiveness of astroglia to LPS, but astroglia did produce a little inducible nitric oxide synthase (iNOS) in the presence of microglia-secreted cytokines [29]. Thus, astroglia might contribute a little IL-10 in the mixed glial cultures.

### 3.2. Early-Phase Released Picomolar IL-10 Protein Exerts Potent Effects on the Expression of Proinflammatory Cytokines

**Figure 2:**
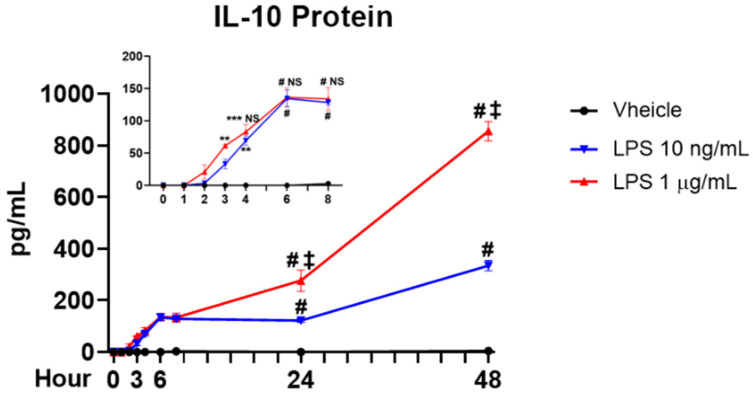
LPS-induced microglial IL-10 protein releases occur in a rapid manner. Supernatant levels of IL-10 were measured by ELISA at 1 h, 2 h, 3 h, 4 h, 6 h, 8 h, 24 h, and 48 h after treatment of LPS at 10 ng/mL or 1 µg/mL. The inset showed the expanded view of the same data within 8 h. Data were from 3 independent experiments. ** *p* < 0.01, *** *p* < 0.001 and # *p* < 0.0001 compared to vehicle group. NS (no significance) and ‡ *p* < 0.0001 compared with LPS 10 ng/mL group. Two-way ANOVA followed by Bonferroni post hoc multiple comparison test.

To determine whether LPS-induced early increase in IL-10 mRNA levels was translated to elevated production and release of IL-10, a detailed time course study was performed at 1 h, 2 h, 3 h, 6 h, 8 h, 24 h, and 48 h after LPS treatment. A significant increase in supernatant IL-10 around 60 pg/mL (equivalent to 3.3 picomolar) was first detected at 3 h in the high dose LPS group (Figure 2 inset). A similar significant increase in IL-10 was found at 4 h in the low dose LPS group (Figure 2 inset). After the initial increase, the supernatant levels of IL-10 reached a plateau lasting for an extended period with no significant differences between low and high LPS dose groups from 4 h to 8 h (Figure 2 inset). A dose-related late-phase increase in IL-10 was observed during the period of 24 h to 48 h showing the high dose LPS triggered twice amounts of IL-10 than that of the low dose LPS group (Figure 2).

**Figure 3:**
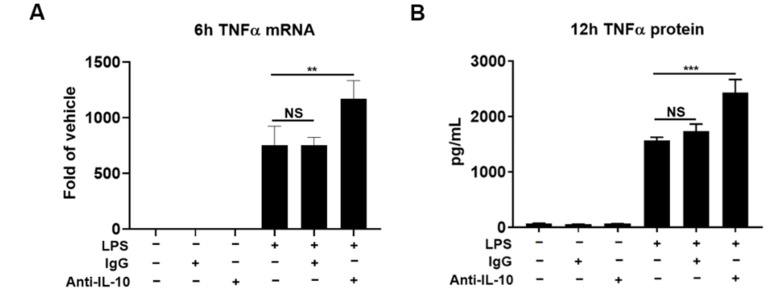
Neutralization of IL-10 enhances LPS-induced increase in TNFα mRNA and protein levels. Following the treatment of with LPS (1 µg/mL) with or without co-treatment of IgG or anti-IL-10 antibody (2.5 µg/mL) in cultures, levels of TNFα mRNA at 6 h point were assessed by qPCR (**A**), and supernatant TNFα protein concentrations were measured at 12 h point by ELISA (**B**). Results were from 3 independent experiments. ** *p* < 0.01 and *** *p* < 0.001 compared to the LPS group. One-way ANOVA followed by Bonferroni post hoc multiple comparison test.

Since the amount of early-released IL-10 by LPS was at picomolar concentrations, we investigated whether this minute concentration of IL-10 could play a role in modulating the expression of proinflammatory cytokine. Supernatant IL-10 protein was depleted by IL-10 neutralizing antibody. TNFα mRNA and protein levels were measured at 6 h and 12 h after treatment, respectively. These two time points were chosen to allow enough time to observe the effect of IL-10 on both TNFα mRNA (6 h) and translated protein (12 h) because the early-released IL-10 was detectable significantly from 3 h after treatment (Figure 2 inset). The results showed that TNFα mRNA levels in the LPS plus IL-10 neutralizing antibody group were higher than those in the LPS alone group (Figure 3A). Furthermore, IL-10 neutralizing antibody led to more TNFα protein release induced by LPS at 12 h (Figure 3B). These results indicate that IL-10 released at the early-phase exerts an inhibitory influence on the expression of TNFα. We also examine IL-1β mRNA levels at the same time points as TNFα mRNA after neutralizing IL-10, but no significant difference was observed (data not shown). Measurements at longer time points are needed to observe increases of IL-1β mRNA compared with TNFα mRNA (Figure 1A,B), which might be the reason why the IL-1β assay at this time point did not show the positive result.

**Figure 4:**
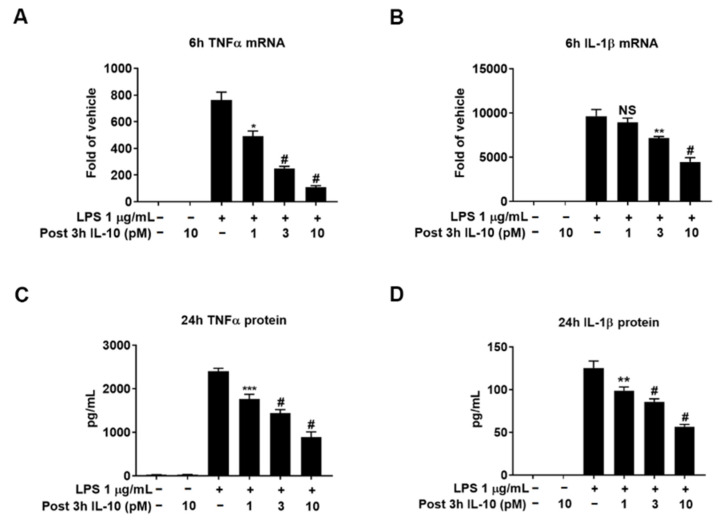
Picomolar IL-10 exerts potent inhibition on the expression of proinflammatory cytokines. Relative TNFα and IL-1β mRNA levels at 6 h were detected in cell cultures by qPCR with the addition of recombinant IL-10 at 1, 3, and 10 pM 3 h after LPS at 1 µg/mL treatment (**A** and **B**). Supernatant protein concentrations of TNFα and IL-1β were measured by ELISA with the same treatment (**C** and **D**). Data were from 3 independent experiments. NS, * *p* < 0.05, ** *p* < 0.01, *** *p* < 0.001, and # *p* < 0.0001 compared with the LPS group. One-way ANOVA followed by Bonferroni post hoc multiple comparison test.

We further investigated whether adding picomolar exogenous IL-10 protein to cultures would reduce LPS-elicited increases in the expression of proinflammatory cytokines. To mimic conditions of endogenous production of IL-10 (Figure 2), recombinant IL-10 was post-treated at 3 h after LPS in extremely low concentrations (1, 3, and 10 pM). The results showed that the expression TNFα and IL-1β mRNA at 6 h was dose-dependently decreased in the presence of picomolar IL-10 (Figure 4A,B). Similarly, protein levels of these proinflammatory cytokines at 24 h were also significantly lowered by the same treatment (Figure 4C,D). These data clearly demonstrated the potent inhibitory function of the early-phase released IL-10 on the LPS-elicited inflammatory process.

### 3.3. Activation of β2-Adrenergic Receptor by Salmeterol Potentiates Early-Phase but Not Late-Phase of IL-10 Expression

Salmeterol is a highly selective, long-acting β2-adrenergic receptor agonist with anti-neuroinflammatory activity. A recent report from Dr. Flood’s laboratory indicates that salmeterol enhances LPS-elicited IL-10 production in BV2 microglia cells and mediates the conversion from proinflammatory to anti-inflammatory phenotype via the MAPK-CREB pathway [27]. Our finding on the LPS-elicited bimodal release of IL-10 offered an opportunity to investigate whether the enhancement of IL-10 by salmeterol depends on the phase-specificity of IL-10 expression and which phase(s) of IL-10 is essential for the anti-inflammatory effect of β2-adrenergic signaling.

**Figure 5:**
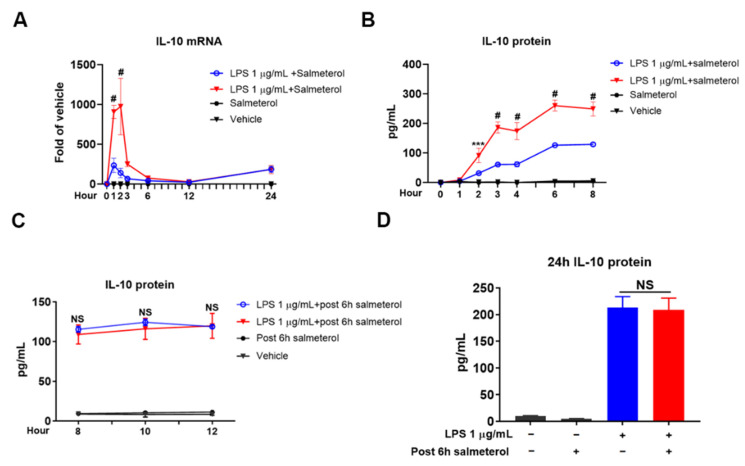
Salmeterol potentiates early-phase but not late-phase IL-10 induction. Relative IL-10 mRNA in cell cultures was measured by qPCR at 1 h, 2 h, 3 h, 6 h, 12 h, and 24 h after treatment of LPS at 1 µg/mL with or without the presence of salmeterol at 10^−9^ M (**A**). Results were from 3 independent experiments. # *p* < 0.0001 compared to LPS alone group. Supernatant IL-10 protein levels were detected by ELISA at 1 h, 2 h, 3 h, 4 h, 6 h, and 8 h after treatment of LPS at 1 µg/mL with or without salmeterol at 10^−9^ M (**B**). Data were from 3 independent experiments. *** *p* < 0.001 and # *p* < 0.0001 compared with LPS alone group. Supernatant IL-10 protein concentrations were assessed by ELISA at 8 h and 10 h (**C**) and 24 h (**D**) after treatment of LPS alone (1 µg/mL) or addition of salmeterol (10^−9^ M) at 6 h after LPS. Results were from 3 independent experiments. NS compared with LPS plus post 6 h salmeterol group. Two-way ANOVA (for **A**–**C**) or one-way ANOVA (for **D**) followed by Bonferroni post hoc multiple comparison test.

Salmeterol alone had no impact on the production of IL-10 but greatly potentiated the effect of LPS. Salmeterol significantly increased early-phase IL-10 mRNA expression within 1–3 h (Figure 5A). Consequently, IL-10 protein levels in the presence of salmeterol were 2–3 folds higher compared with those of LPS alone (Figure 5B). Interestingly, salmeterol failed to upregulate late-phase expression of IL-10 mRNA between 6 and 24 h (Figure 5A) and at 48 h (Supplementary Figure S1a) after LPS stimulation. In another experiment, we found that post-treated salmeterol at 6 h after LPS exerted no potentiating effect on the production of IL-10 at the later hours (Figure 5C,D). Consistently, post-treatment of salmeterol at 48 h after LPS was not able to affect IL-10 mRNA transcription (Supplementary Figure S1b). These data indicate that the potentiating effect of salmeterol on IL-10 production is phase-specific.

### 3.4. Early-Phase Released IL-10 Is Crucial for the Anti-Inflammatory Function of Salmeterol in Both Repressing Proinflammatory Response and Promoting Arg1 Expression

**Figure 6:**
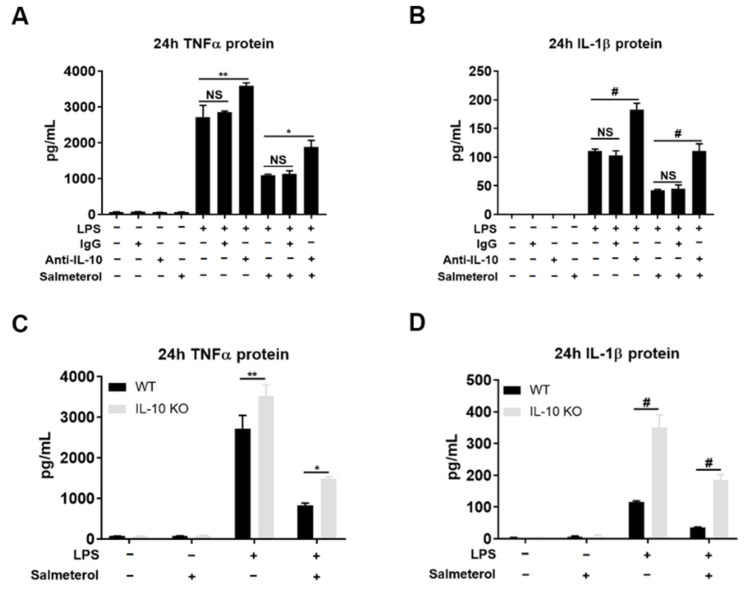
IL-10 deficiency impairs inhibitory potency of salmeterol on LPS-elicited increase in proinflammatory cytokines. Supernatant protein concentrations of TNFα (**A**) and IL-1β (**B**) were detected by ELISA at 24 h after treatment of LPS 1 µg/mL, IgG 2.5 µg/mL, anti-IL-10 antibody 2.5 µg/mL, and salmeterol 10^−9^ M as indicated. Supernatant levels of TNFα (**C**) and IL-1β (**D**) were measured by ELISA at 24 h after LPS 1 µg/mL, and salmeterol 10^−9^ M treatment as indicated in wildtype and IL-10 KO mixed glial cultures. Data were from 3 independent experiments. * *p* < 0.05, ** *p* < 0.01, and # *p* < 0.0001. One-way ANOVA (for picture **A** and **B**) or Two-way ANOVA (for pictures **C** and **D**) followed by Bonferroni post hoc multiple comparison test.

The finding that salmeterol potentiated IL-10 production in a phase-specific manner led us to investigate the role of early-phase released IL-10 in modulating the anti-inflammatory function of salmeterol. For this purpose, we examined the inhibitory potency of salmeterol on the LPS-elicited expression of proinflammatory cytokines by eliminating IL-10 from the cultures by either neutralizing IL-10 antibodies or primary mixed glial cultures prepared from IL-10-deficient mice. LPS-elicited production of proinflammatory cytokine TNFα and IL-1β was greatly suppressed by salmeterol at 24 h after treatment, but the inhibitory effects of salmeterol on these cytokines were significantly weakened in IL-10 neutralizing antibody-treated cultures (Figure 6A,B). Similarly, genetic deletion of IL-10 also diminished the efficacy of salmeterol in reducing the release of proinflammatory cytokine in the cell cultures (Figure 6C,D). Since salmeterol does not influence the production of late-phase IL-10, these data clearly demonstrate the essential role of early-phase released IL-10 in mediating the anti-inflammatory function of salmeterol.

**Figure 7:**
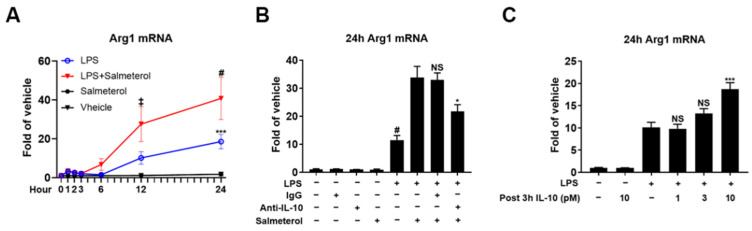
IL-10 involves salmeterol-upregulated Arg1 mRNA expression in the presence of LPS. Relative Arg1 mRNA in cell cultures were assessed by qPCR at 1 h, 2 h, 3 h, 6 h, 12 h, and 24 h after LPS 1 µg/mL and salmeterol 10^−9^ M treatment as indicated (**A**). Results were from 3 independent experiments. *** *p* < 0.001 compared to vehicle group; ‡ *p* < 0.001 and # *p* < 0.0001 compared to LPS group. Two-way ANOVA followed by Bonferroni post hoc multiple comparison test. Relative Arg1 mRNA was measured in cell cultures by qPCR at 24 h after LPS 1 µg/mL, salmeterol 10^−9^ M, and anti-IL-10 antibody treatment as indicated (**B**). Results were from 3 independent experiments. NS, * *p* < 0.05 and # *p* < 0.0001 compared with LPS plus salmeterol group. One-way ANOVA followed by Bonferroni post hoc multiple comparison test. Relative Arg1 mRNA was detected in cell cultures by qPCR at 24 h after LPS 1 µg/mL, with post-3 h treatment of recombinant IL-10 protein at different concentrations as indicated (**C**). Results were from 3 independent experiments. NS and *** *p* < 0.001 compared to LPS group. One-way ANOVA followed by Bonferroni post hoc multiple comparison test.

LPS induced a slow increase in the expression of Arg1 mRNA, which was markedly enhanced between 12 h and 24 h after LPS by the co-treatment with salmeterol (Figure 7A). However, the potentiating activity of salmeterol was significantly reduced by the addition of an IL-10 neutralizing antibody (Figure 7B). Moreover, Arg1 mRNA expression at 24 h was upregulated significantly by post addition of ten picomolar IL-10 after LPS but not by IL-10 alone (Figure 7C). Therefore, IL-10 at the early stage is required for the optimal efficacy of salmeterol in amplifying Arg1 induction.

## 4. Discussion

**Figure 8:**
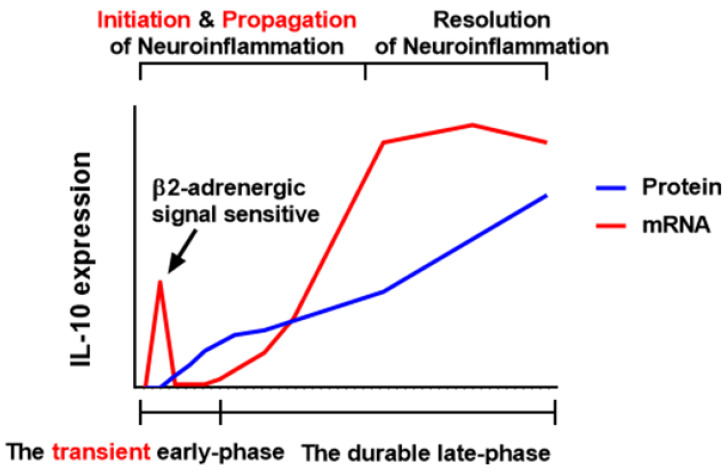
Schematic drawing the relationship between the stage of neuroinflammation and IL-10 expression. Upon TLR4 activation, IL-10 expresses rapidly other than in a previously reported delayed manner. Early-expressed IL-10 releases at very low levels compared to the late-phase but exert powerful regulatory effects on LPS-elicited neuroinflammation. A unique bimodal transcriptional fashion of IL-10 underlies the temporal pattern of IL-10 secretion. The transient early-phase of IL-10 mRNA induction grants the quick release of a low quantity of IL-10 to precisely modulate the initiation and propagation of neuroinflammation while the durable late-phase of IL-10 mRNA transcription sustains the delayed production of a high quantity of IL-10 to facilitate the resolution of neuroinflammation. The β2-adrenergic signal potentiates the early- but not late-phase of IL-10 induction. It suggests that the phase-specificity should be considered when investigating regulation mechanisms of a certain compound for microglial IL-10 expression.

This study demonstrated a bimodal pattern (two-phase expression) of LPS-elicited microglial IL-10 mRNA expression and critical roles of early-released IL-10 in modulating neuroinflammation, overturning the previous notion that microglial IL-10 produces and functions in a delayed manner. The early-released IL-10 in the current study offered a possible mechanism underlying rapidly aggravated neuroinflammation occurring in IL-10^−/−^ mouse brains reported by our lab [24]. The phase-specificity of IL-10 induction may be considered when investigating regulation mechanisms of a certain compound for microglial IL-10 expression. A schematic drawing illustrating the expression and functional role of IL-10 in modulating various stages of LPS-elicited neuroinflammation was shown in Figure 8.

### 4.1. The Critical Role of Early-Phase Released IL-10 in Regulating Neuroinflammation from the Initial Stage

Reports in vitro and in vivo indicate that IL-10 is released from microglia in a delayed manner [12,13,17,18,19,20,21]. Here, we found that microglial IL-10 protein can be produced rapidly and played critical roles in neuroinflammation (Figure 2, Figure 3 and Figure 4). The finding of early-phase released IL-10 in the present study has shown several novel anti-inflammatory functions during LPS-elicited neuroinflammation: (1) Early-released IL-10 is responsible for modulating mRNA levels of proinflammatory cytokines, such as TNFα and IL-1β, at the early hours after LPS treatment. By fastening the degradation of mRNA, IL-10 can effectively reduce the steady state concentrations of mRNA levels of these cytokines. Our results indicate that removal of early-released IL-10 significantly enhanced TNFα mRNA levels after LPS treatment (Figure 3). Adding picomolar exogenous IL-10 3 h after LPS decreased mRNA levels of TNFα and IL-1β (Figure 4A,B). By contrast, IL-10 released at the late-phase (24–48 h after LPS) was likely too late to serve this function, since, by these time points, most mRNA levels for these proinflammatory cytokines have returned to control values; (2) Demonstration of the high potency of IL-10 in reducing proinflammatory cytokine mRNA levels. Although IL-10 in ng/mL concentrations was often used in most previous reports to show its anti-inflammatory function [6,31], we demonstrated that the addition of minute amounts (1–10 pM) of exogenous IL-10, which is close to the amount of IL-10 released at the early-phase, repressed LPS-elicited increase in mRNA levels of major proinflammatory cytokines. The endogenous concentrations of IL-10 after LPS were about 60 pg/mL (equivalent to 3.3 picomolar) at 3 h when the exogenous IL-10 was added. The significant effects of minute amounts of exogenous IL-10 indicate that, at the initiation of neuroinflammation, the cells are highly sensitive to the minute amount of IL-10, and the quantity of IL-10 released is tightly controlled to avoid overreacted or compromised responses. These concentrations are even below the reported Kd values (50–200 pM) of IL-10 in JY and MC/9 cell lines [32]. A recent article has demonstrated low concentrations but high potency of IL-10 in vivo. Peripheral LPS-stimulated brain produced a very low amount of IL-10 (1–4 pg/mg brain protein), but the minute IL-10 confers significant microglial neuroprotective abilities to alleviate multiply neuropathies [33]. Of note, mixed glial cultures contained both microglia and astroglia, as mentioned in the Materials and Methods. Astroglia-enriched cultures did respond to LPS stimulation as indicated by our previous study [26], but they might still contribute a small amount of IL-10, TNFα, and IL-1β in the mixed glial cultures. However, this does not affect the relevance of the possible protective action of the released IL-10.

### 4.2. A Bimodal LPS-Elicited Increase in Expression of Microglial IL-10 mRNA in Mouse Primary Glial Cultures

Two separate and distinct stages of IL-10 mRNA transcription were revealed by a detailed mRNA analysis (Figure 1). Differences in the IL-10 mRNA profile were observed between these two stages. The early increase in IL-10 mRNA is modest, quick onset (1–2 h), and short-lived. By contrast, a profound and long-lasting increase was found in the late-phase expression. The rapid increase in early-phase IL-10 mRNA seems associated with the innate response to LPS via TLR4 activation, such as the transcription of proinflammatory cytokines at the initial stage of neuroinflammation. By contrast, late-stage IL-10 mRNA transcription might be linked with the secondary reaction to preceding inflammatory responses, such as the release of inflammatory mediators [17,19,34] or damage-associated molecules [35]. Furthermore, β2-adrenergic receptor activation by salmeterol upregulates early-phase, but not late-phase, IL-10 production (Figure 5 and Supplementary Figure S1), implying that production of IL-10 at early and late stages is regulated by different cellular signalings.

### 4.3. The Pivotal Role of Quick-Primed IL-10 in β2-Adrenergic Receptor Antagonists-Modified Neuroinflammation

The anti-inflammatory effect of β2-adrenergic signaling was utilized as a therapeutic target for neuroinflammation-related disorders [26]. Recently, salmeterol was found to upregulate IL-10 production and accelerate the expression of M2 marker Arg1 in LPS-activated microglial BV2 cells [27]. We have obtained similar results in primary microglial cultures showing salmeterol potentiated LPS-elicited production of IL-10 (Figure 5A,B). We extended these findings to uncover more detailed regulatory mechanisms. Our data indicate that salmeterol potentiated only early-, but not late-released IL-10 (Figure 5C,D). In addition, both proinflammatory cytokine inhibition and Arg1 enhancement by salmeterol were hampered by the genetic or pharmacological removal of IL-10 (Figure 6 and Figure 7). Thus, the early-phase IL-10 is required for optimal effects of salmeterol in exerting its anti-inflammatory actions. Since the increased IL-10 protein was released at 2 h later in the presence of salmeterol (Figure 5B) and it seemed that Arg1 expression responded slowly to IL-10 stimulation, the increment of Arg1 mRNA by salmeterol treatment occurred at a later stage (12 h and 24 h later) but not early stage (Figure 7A). A critical role of early release IL-10 in facilitating the transition of microglia to a more anti-inflammatory phase is further supported by the result showing high potency of IL-10 in upregulating M2 marker Arg1 mRNA levels in LPS-stimulated cell cultures (Figure 7). It is important to point out that astroglia also expresses β2-adrenergic receptors that modulate the inflammatory response upon TNFα stimulation [36]. Hence, astroglia might participate in the effect of salmeterol in the mixed glial cultures.

## 5. Conclusions

This study reveals an unexpected early release of LPS-induced microglial IL-10 in vitro, which serves dual function at picomolar levels in reducing the expression of proinflammatory cytokines and promoting the transition of microglial phenotype to a more anti-inflammatory phase. Our study suggests the appropriate initial degree of neuroinflammation is tightly controlled by early-released IL-10, providing new insights into anti-inflammatory mechanisms-mediated neuroimmune homeostasis.

## Data Availability

All data generated or analyzed in this study are included in this article.

## Supplementary Materials

The following are available online at https://www.mdpi.com/article/10.3390/cells10092173/s1, Figure S1: Salmeterol does not potentiate late-phase IL-10 induction.
