# Supplementary material for: Early-Released Interleukin-10 Significantly Inhibits Lipopolysaccharide-Elicited Neuroinflammation In Vitro
Source: Cells. 2021 Aug 24;10(9):2173. doi: 10.3390/cells10092173 (PMC8466025; doi:10.3390/cells10092173)
Supplement: Supplementary file 1 [file cells-10-02173-s001.zip › cells-1303449-supplementary.pdf]

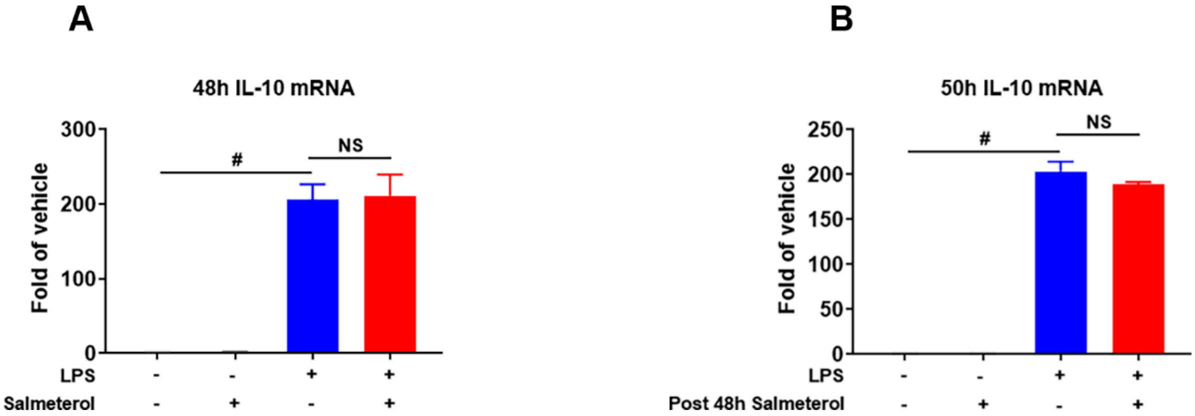

Supplement Figure S1. Salmeterol does not potentiate late-phase IL-10 induction. Relative IL-10 mRNA in cell cultures was evaluated by qPCR at 48 h after treatment of LPS at 1 μg/mL with or without 10-9 M salmeterol (**A**). Relative IL-10 mRNA in cell cultures was detected by qPCR at 50 h after treatment of LPS alone (1 μg/mL) or addition of 10-9 M salmeterol at 48 h after LPS (**B**). Results were from 3 independent experiments. #$p < 0.0001$ compared to vehicle group and NS compared to LPS plus salmeterol group. One-way ANOVA followed by Bonferroni post hoc multiple comparison test.
